# Nrf2 Levels in Human Semen and Spermatozoa: Association with F_2_-Isoprostanes as Markers of Lipid Peroxidation

**DOI:** 10.3390/ijms27115079

**Published:** 2026-06-04

**Authors:** Elena Moretti, Cinzia Signorini, Laura Liguori, Elena Leoni, Giulia Collodel

**Affiliations:** Department of Molecular and Developmental Medicine, University of Siena, 53100 Siena, Italy; elena.moretti@unisi.it (E.M.); laura.liguori@student.unisi.it (L.L.); elena.leoni@student.unisi.it (E.L.); giulia.collodel@unisi.it (G.C.)

**Keywords:** human semen, Nrf2 pathway, F_2_-Isoprostanes, varicocele, urogenital infections

## Abstract

Despite the recognized role of oxidative stress (OS) in sperm function, limited data exist on Nuclear factor-E2-related factor 2 (Nrf2) pathway modulation in relation to reliable oxidative damage markers in human semen. In this study, 79 semen samples were collected from men undergoing semen analysis and grouped as varicocele (V, no. 22), urogenital infections (UI, no. 23), unknown fertility status without pathologies (UFS, no. 15), and fertile controls (F, no. 19). After semen analysis, ELISA were used to quantify F_2_-Isoprostane (F_2_-IsoPs) level, a marker of lipid peroxidation, and Nrf2 in seminal plasma and spermatozoa. The Nrf2 pathway (Keap1, Nrf2, Bach1, HO-1) was assessed in spermatozoa by qRT-PCR. Seminal plasma and sperm Nrf2 positively correlated with F_2_-IsoPs (*p* < 0.001) and negatively with sperm vitality. Sperm Nrf2 also inversely correlated with progressive motility (*p* < 0.05). Seminal F_2_-IsoP levels were lower in F than in the other groups. Sperm Nrf2 was significantly lower in F versus V and UI (*p* < 0.001) and UFS (*p* < 0.05), while seminal plasma Nrf2 levels did not differ among groups. qRT-PCR suggested Nrf2 pathway activation mainly in V and UI, consistent with increased OS. Elevated F_2_-IsoPs, a marker of poor sperm quality, and sperm Nrf2 could suggest OS-driven Nrf2 activation, providing complementary biomarkers of oxidative status in male reproductive health.

## 1. Introduction

Increasing evidence suggests that the common causes of reduced semen quality, such as varicocele, urogenital infection [1], and idiopathic infertility [2,3], share a pathogenic mechanism linked to oxidative stress (OS) and inflammation.

This condition is characterized by an imbalance between elevated levels of reactive oxygen species (ROS) and the antioxidant defense system [4,5], resulting in alterations of cellular metabolism and damage to critical cellular and subcellular structures [6,7,8]. OS is not detected by routine semen analysis and, even when routine semen analysis appears as “normal,” it can cause several functional sperm damages. Normal semen was defined according to WHO reference values [9], based on the assessment of semen volume, sperm concentration, progressive motility, normal morphology, and vitality. In particular, the lower reference limits corresponding to the 5th percentile of a fertile population were used, including thresholds of ≥30% for progressive motility, ≥4% for normal morphology, and ≥54% for sperm vitality to discriminate between normal and abnormal semen. Therefore, the OS assessment is assuming an increasingly important role in the evaluation of male human fertility. In this regard, F_2_-Isoprostanes (F_2_-IsoPs), originating from the non-enzymatic, free-radical-induced peroxidation of arachidonic acid, are emerging as reliable biomarkers of OS, reflecting lipid peroxidation both systemically [10] and in human semen [11], where elevated levels indicate oxidative damage to sperm membranes.

ROS acts as a major trigger of the Nuclear factor-E2-related factor 2 (Nrf2) signaling pathway; Nrf2 is an endogenous transcription factor that activates the cells’ antioxidant pathways, preventing redox imbalances [12]. Briefly, under homeostatic conditions, Kelch-like ECH-associated protein 1 (Keap1) binds Nrf2, promoting its ubiquitination and proteasomal degradation; thus, BTB and CNC homology 1 (Bach1), linked to small Musculoaponeurotic Fibrosarcoma (sMAF), suppresses antioxidant response element (ARE)-driven gene transcription. During cellular stress, the high ROS concentrations disrupt the interaction between Keap1 and Nrf2 and allow stabilized or newly synthesized Nrf2 [13] to accumulate and translocate into the nucleus. In the nucleus, Nrf2 replaces Bach1 and heterodimerizes with sMAF, inducing gene expression of several enzymes with antioxidant activity, including heme oxygenase-1 (HO-1) and glutathione peroxidase 4 [14,15].

Nrf2 seems to play a central role in the pathogenesis of both female and male reproductive disorders [16,17], and substantial evidence suggests a critical role of Nrf2 in protecting spermatogenesis from oxidative damage.

Male fertility/infertility cannot be reliably determined merely through the routine evaluation of semen parameters, which, however, represents the first-line intervention for evaluating male fertilizing capacity. It is important to underline that the WHO reference ranges for sperm count, motility, and morphology do not accurately differentiate between fertile and infertile men [18].

The study was based on the hypothesis that OS and lipid peroxidation in spermatozoa are associated with variation in the Nrf2 antioxidant signaling pathway. Specifically, it was hypothesized that increased levels of lipid peroxidation markers, such as F_2_-IsoPs, would be related to different Nrf2 levels in seminal plasma and spermatozoa.

To investigate this hypothesis, routine semen parameters were first evaluated according to WHO guidelines [9], including semen volume, sperm concentration, motility, morphology, and vitality. F_2_-IsoPs levels were then measured in seminal plasma as indicators of lipid peroxidation, Nrf2 protein levels were assessed in seminal plasma and spermatozoa, while qRT-PCR analysis was performed to evaluate the expression of key genes involved in the Nrf2 signaling pathway, including *NFE2L2* (Nrf2), *KEAP1* (Keap1), *BACH1* (Bach1), and *HMOX1* (HO-1). The expected outcome was that OS conditions would induce changes in the expression of these genes, reflecting activation or dysregulation of the cellular antioxidant defense response. In particular, altered Nrf2 and HO-1 expression, together with modulation of Keap1 and Bach1, were expected to provide insight into the adaptive antioxidant mechanisms associated with sperm oxidative damage.

## 2. Results

Sixty subjects were included in this study: 22 out of 60 were affected by varicocele (group V), 15 UFS subjects without systemic and reproductive pathologies (group UFS), and 23 out of 60 had positive semen culture and were classified as having urogenital infection (group UI): eight had *Enterococcus faecalis*, eight had *Escherichia coli*, four had *Staphylococcus haemolyticus*, two had *Staphylococcus aureus,* and one had *Ureaplasma urealyticum*.

Semen samples of 19 fertile subjects (group F) represented the control group. Variables such as semen parameters, seminal F_2_-IsoPs, and both seminal and sperm cells’ Nrf2 levels were evaluated across all groups (79 subjects). The pH of the semen samples of the studied population was similar and ranged from 7.3 to 7.8. The results of the whole population are reported in Table 1 as medians (IQR).

**Figure 1:**
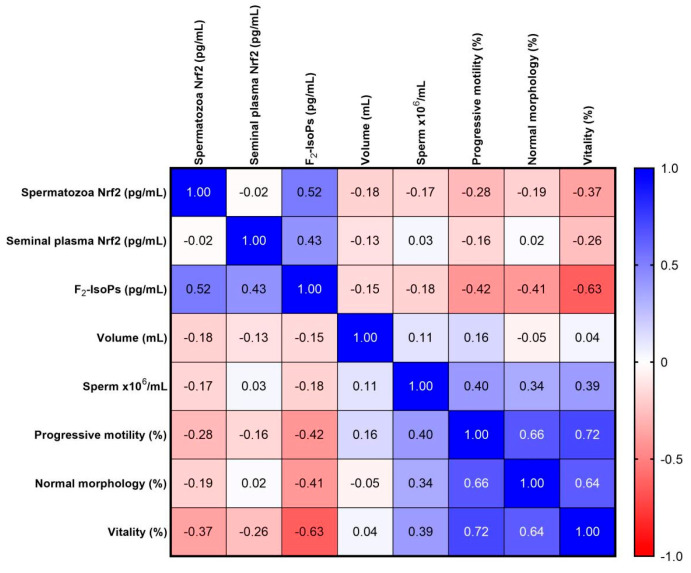
Heat map showing the distribution of values across the analyzed variables. Warmer colors indicate higher values, while cooler colors represent lower values of correlation.

Spearman’s rank correlation coefficient was used to assess the correlations between the studied variables across the entire population of interest. The results are reported in Table 2 and Figure 1. The seminal parameters, such as sperm concentration, motility, morphology, and vitality (Table 2), were positively correlated with each other.

Both seminal plasma and sperm cell Nrf2 levels showed significant positive correlations with F_2_-IsoP levels (*p* < 0.001) and significant negative correlations with vitality (*p* < 0.05 and *p* < 0.001, respectively). Sperm vitality, normal morphology, and progressive motility were negatively correlated with F_2_-IsoP levels (*p* < 0.001). Sperm Nrf2 levels are negatively correlated with progressive motility (*p* < 0.05).

The same correlation between variables can be powerfully visualized in a heat map (Figure 1), in which color gradients range from cool (low values) to warm tones (high values), indicating the direction and strength of correlations.

The considered variables of the different groups (F, V, UI, UFS) and the statistics are reported in Table 3.

Fertile subjects exhibited better semen parameters than all other groups. Specifically, the F group showed significantly higher sperm concentration than the UFS group and higher progressive motility, normal morphology, and vitality compared with the other analyzed groups (Table 3).

The levels of F_2_-IsoPs (Table 3) were lower in the seminal plasma of fertile subjects than those measured in the seminal plasma of patients with varicocele and urogenital infections (*p* < 0.001), and the UFS group (*p* < 0.05). In addition, F_2_-IsoP concentrations were significantly increased in the seminal plasma of the V group compared to the UFS group (*p* < 0.05).

Seminal plasma Nrf2 levels (Table 3) did not differ significantly among groups. However, levels were higher in the V (38.04 pg/mL) and UI (31.25 pg/mL) groups than those observed in the F (23.58 pg/mL) and UFS (16.30 pg/mL) groups.

Spermatozoa Nrf2 concentrations (Table 3) were significantly increased in V (142.70 pg/mL; *p* < 0.001), UI (183.40 pg/mL; *p* < 0.001), and UFS groups (88.31 pg/mL; *p* < 0.05), compared with those measured in sperm cells of fertile men (38.80 pg/mL). Notably, the highest spermatozoa Nrf2 levels were observed in the UI and V groups. Gene expression related to the Nrf2 pathway was evaluated in six randomly selected spermatozoa samples for each group. The considered variables of the different groups (F, V, UI, UFS) and the statistics are reported in Table 3.

**Figure 2:**
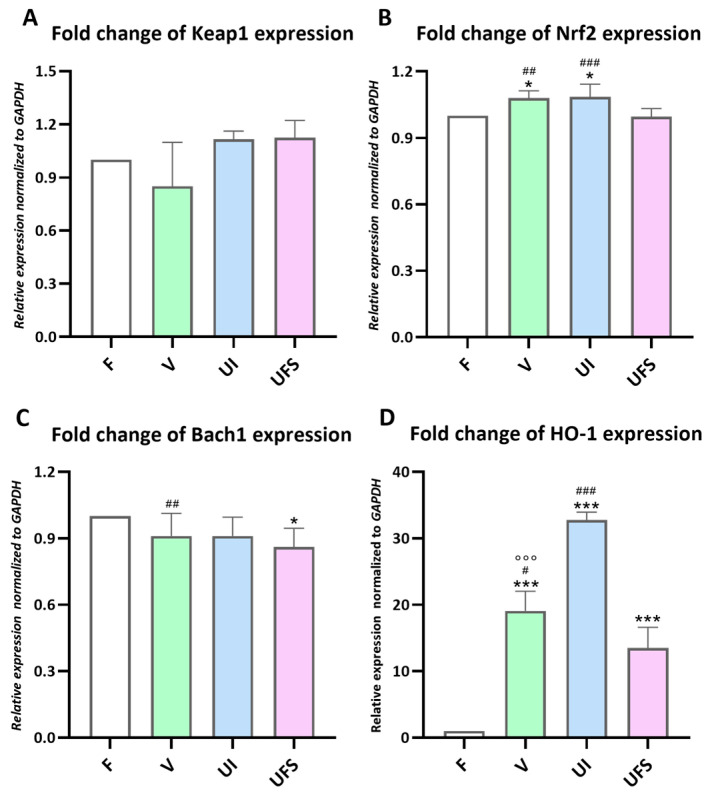
Fold change in Keap1 (**A**), Nrf2 (**B**), Bach1 (**C**), and HO-1 (**D**) expression normalized to *GAPDH* in six randomly selected spermatozoa samples for each group (a total of 24 samples). * *p* < 0.05; *** *p* < 0.001 vs. fertile (F) group; # *p* < 0.05; ## *p* < 0.01; ### *p* < 0.001 vs. unknown fertility status (UFS) group; °°° *p* < 0.001 vs. urogenital infection (UI) group.

Keap1 (Figure 2A) levels did not differ significantly among the groups. Nrf2 (Figure 2B) was overexpressed in both the V and UI groups compared with fertile subjects (both *p* < 0.05) and with the UFS group (V: *p* < 0.01; UI: *p* < 0.001). Bach1 (Figure 2C) was downregulated in all groups compared with fertile subjects and was significantly lower in the UFS group than in both the F (*p* < 0.05) and the V groups (*p* < 0.01).

HO-1 expression (Figure 2D) was significantly higher in the V, UFS, and particularly in the UI groups compared with the F group (*p* < 0.001). Moreover, HO-1 was significantly overexpressed in the UI group compared with both the V and UFS groups (both *p* < 0.001), and it was also higher in the V group than in the UFS group (*p* < 0.05).

## 3. Discussion

OS is widely recognized as a major contributor to sperm dysfunction and male infertility, due to its detrimental effects on sperm membrane lipids, DNA integrity, and overall sperm quality [19]. In this context, the Nrf2 represents a master regulator of the cellular antioxidant defense system, orchestrating the transcription of genes involved in redox homeostasis. Thus, Nrf2, a redox-sensitive transcription factor that regulates OS by transcribing mRNA for antioxidant enzymes, represents a molecule of extraordinary relevance in male reproduction [20] as supported by the development of Nrf2^−/−^ mice [21], which also exhibit dysregulation of mechanisms preventing ferroptosis [22].

Despite the well-established role of OS in semen physiology, limited information is available on the behavior of the Nrf2 signaling pathway in relation to reliable biomarkers of oxidative damage in human semen. Among these, F_2_-IsoPs are considered robust and specific indices of lipid peroxidation and OS [11]. Therefore, the present study provides novel insights by evaluating the Nrf2 pathway in relation to seminal F_2_-IsoP levels in subjects undergoing semen analysis, highlighting the importance of integrating molecular antioxidant pathways with validated OS markers in the assessment of male reproductive health. Our cohort derives from a real-world setting within a sperm analysis laboratory, where subjects are not exclusively evaluated in the context of attempting conception, but rather undergo semen assessment for a variety of clinical or preventive reasons. In this context, the population is more representative of routine clinical practice in andrology.

Within the field of male reproduction, numerous studies using animal models have demonstrated that experimentally induced testicular damage, caused by various toxic agents, can be counteracted by the administration of compounds capable of activating the Nrf2 pathway, resulting in an improvement of spermatogenesis and overall reproductive outcomes [23,24,25,26,27].

Using a similar approach, Jannatifar and colleagues [28], in a randomized, blinded clinical trial study, treated 50 asthenoteratozoospermic infertile patients with an oral supplementation of N-acetyl-L-cysteine and found that this compound protected against OS by increasing Nrf2 expression.

The data obtained in this study demonstrated strong positive correlations between F_2_-IsoP levels, a marker of lipid peroxidation, and the concentrations of Nrf2 measured both in seminal plasma and spermatozoa. These two relationships have different significances.

Nrf2 itself, being a transcription factor, is not actively secreted as a soluble protein in seminal plasma under normal conditions. So, seminal plasma Nrf2 could be interpreted as a marker of OS, and increased Nrf2 levels in seminal plasma may reflect damage occurring in the prostate and seminal vesicles, producing the vast majority of seminal plasma, rather than protection. This observation agrees with a study on semen from patients with leukocytospermia that reported increased Nrf2 expression in seminal plasma samples associated with inflammation and OS, suggesting that the presence of Nrf2 may be linked to pathological processes rather than to a physiological antioxidant activity [29].

On the other hand, the concomitant increase in seminal plasma F_2_-IsoP levels and Nrf2 concentrations in spermatozoa suggests that, during spermatogenesis under oxidative conditions, germ cells upregulate the Nrf2 pathway as they mature.

Sperm Nrf2 levels seem to be less associated with conventional semen parameters and more closely related to lipid peroxidation levels. Indeed, subjects with varicocele and urogenital infections showed both high seminal F_2_-IsoP levels and elevated sperm Nrf2 concentration, suggesting a reactive upregulation of Nrf2 during spermatogenesis in response to the presence of ROS, as demonstrated also by a qRT-PCR gene expression study.

It is well known that OS is a common denominator and has a role in the pathogenesis of varicocele [30] and urogenital infections [31].

In the presence of varicocele and urogenital infections, the upregulation of sperm Nrf2 (a marker of antioxidant defense) during spermatogenesis, in response to increased ROS levels and the resulting lipid peroxidation, is concomitant with the relatively preserved semen parameters, which do not appear to be markedly altered in these subjects with reproductive pathologies and with unknown reproductive potential.

Therefore, the increase in sperm Nrf2 and semen F_2_-IsoP levels in response to excessive ROS production may indicate activation of compensatory antioxidant mechanisms in spermatozoa, which still appear insufficient to prevent lipid peroxidation and impaired sperm motility.

A negative correlation between sperm Nrf2 levels and progressive sperm motility was observed in our studied population. In contrast to our findings, previous studies have reported a downregulation of Nrf2 in patients with asthenozoospermia and oligozoospermia [22,32,33]. Clearly, patient selection plays a fundamental role, and these studies did not evaluate oxidative damage levels, which represents a major limitation, as ROS are primary activators of the Nrf2 signaling pathway [15]. Importantly, asthenozoospermia and oligozoospermia are not invariably associated with OS, but can be due to other structural and biochemical alterations of spermatozoa. In addition, functional nucleotide polymorphisms in the Nrf2 promoter region have been identified in idiopathic asthenozoospermic and oligoasthenozoospermic patients compared with controls. These variants have been associated with altered Nrf2 mRNA expression and/or reduced expression of antioxidant enzymes in spermatozoa [34] and with sperm DNA damage [35].

The group of patients with unknown fertility status and without reproductive pathologies, the UFS group, is unfortunately poorly defined; however, each patient exhibits at least one seminal parameter below the lower reference limit reported in WHO guidelines [9]. In this group as well, F_2_-IsoP levels are higher than in controls, indicating the oxidative-induced fatty acid damage, concomitant with an upregulation of sperm Nrf2, although to a lesser extent than in subjects with varicocele and urogenital infections.

The relevance of F_2_-IsoP levels in discriminating physiological and pathological values of these compounds in human semen was previously indicated by Moretti and colleagues [36], with reference to the total amounts of F_2_-IsoPs (determined as both molecules present in free form in biological fluids and forms esterified to fatty acids). Accordingly, sperm Nrf2 levels could reflect the physiological basal activation of the oxidative fatty acid pathway.

The study of gene expression of the Keap1–Nrf2 signaling pathway confirmed these observations. While it is well established that spermatozoa are translationally inactive and do not perform protein synthesis, it is equally well documented that they contain a diverse population of RNAs, including mRNAs. These transcripts are remnants of spermatogenesis and/or may have functional roles after fertilization [37].

The increased mRNA expression levels of the transcription factor Nrf2, mainly in subjects with varicocele and urogenital infection, agreed with the results obtained by ELISA and with the concomitant downregulation of the repressor Bach1.

In conditions where diseases share a basis of OS, the Nrf2 pathway appears to be activated during spermatogenesis, and the HO-1 gene is also overexpressed, indicating a systemic response that limits and prevents significant damage to sperm cells, as demonstrated by the values of the conventional semen parameters. It is possible, though not certain, that the concentration and activity of HO-1 may also be elevated. HO-1 has been recognized as a protective antioxidant that can be induced by various stressors, including ROS. The upregulation of the HO-1 gene expression in response to different oxidative stimuli represents a key event in the cellular adaptive response. Consuming molecular oxygen under oxidative conditions, it helps limit the generation of ROS, thereby providing indirect cytoprotection [15,38]. The expression pattern of Keap1 did not show any significant differences among groups. However, the regulatory activity of Keap1 is mainly controlled through conformational alterations and post-translational modifications rather than changes in its transcriptional levels. Keap1 is a protein highly enriched in cysteine residues, many of which can be modified by various oxidants; three cysteine residues—C151, C273, and C288—have been demonstrated to play key functional roles. Their modification induces conformational changes in Keap1 that lose the ability to ubiquitinate Nrf2, thereby stabilizing the protein and facilitating its translocation into the nucleus and the activation of the transcription of downstream antioxidant genes [39,40].

In this study, patient selection was not based on infertility criteria, as our primary aim was to investigate the relationship between the Nrf2 pathway and damage induced by the presence of ROS, which, when exceeding physiological levels, can lead to OS. Based on our findings, we suggest that the evaluation of the Nrf2 should be complemented by a marker identifying ROS levels or ROS-induced damages in order to obtain a comprehensive view of this crosstalk. It is also evident that, using this approach, infertile men should be analyzed to determine whether this crosstalk is preserved or, for some reason, disrupted.

This study has some limitations that should be acknowledged. First, no specific spermatozoa purification procedure was performed before RNA extraction. However, during routine semen analysis, none of the seminal samples included in the study showed the presence of leukocytes or epithelial cells according to microscopic evaluation.

In addition, GAPDH expression stability across the fertile, varicocele, urogenital infection, and unexplained infertility groups was not specifically assessed.

The use of GAPDH as a housekeeping gene is widely accepted and has been previously reported in studies on human spermatozoa [41,42]. However, it should be considered that GAPDH expression may show variability, whereas B2M [43] has been reported as one of the most stably expressed reference genes.

## 4. Materials and Methods

### 4.1. Human Semen Samples

Sixty semen samples were obtained from subjects (aged 22–34 years) attending the Department of Molecular and Developmental Medicine, University of Siena (Italy). The study was conducted in accordance with the Declaration of Helsinki and approved by the Ethics Committee of Siena University Hospital (ID: CEAVSE 25612). All participants were fully informed about the study and provided written informed consent prior to enrolment, agreeing to the use of their semen samples exclusively for scientific research purposes.

The study included men with unknown fertility status who underwent semen analysis for routine evaluation.

A full and detailed medical history was obtained for each subject. The eligibility criteria were absence of azoospermia, absence of leukocytospermia, BMI < 25 kg/m^2^, presence of varicocele, and urogenital infections. Exclusion criteria were chronic illnesses, hormonal imbalance, radiotherapy or chemotherapy treatments, current medication use, and intake of oral antioxidant supplements within the previous four months. Individuals with a history of recreational drug use or alcohol consumption were also not selected. Men who smoked more than 10 cigarettes per day were excluded from the study.

The selected participants were categorized into three clinical groups:

- Subjects with unknown reproductive potential with clinically diagnosed varicocele (22 subjects);
- Subjects with unknown reproductive potential with urogenital infections (23 subjects);
- Subjects with unknown fertility status, without known reproductive or systemic pathologies (15 subjects).

The varicocele group included only patients previously evaluated through both physical examination and scrotal color Doppler ultrasonography performed in an external laboratory; individuals with subclinical varicocele were excluded. The bacteriological analyses were provided by the patients, and those with positive semen cultures were completely asymptomatic.

A control group of 19 fertile men (aged 25–36 years, Ethics Committee of Siena University Hospital [ID: CEAVSE 25612]), all of whom had fathered at least one child in the previous three years, was also enrolled. These subjects showed no signs of infection or anatomical abnormalities.

### 4.2. Semen Analysis

Semen samples were collected by masturbation into sterile, non-toxic containers after 2–5 days of sexual abstinence. Following complete liquefaction at 37 °C for 30 min, conventional semen analysis was performed in accordance with the recommendations of the sixth edition of the WHO Manual for the Examination and Processing of Human Semen [9]. Semen volume, pH, sperm concentration, and sperm progressive motility were evaluated. Sperm morphology was assessed using pre-stained Testsimplets^®^ slides (Waldeck GmbH & Co. KG, Münster, Germany), and at least 300 spermatozoa per sample were examined by light microscopy. Sperm vitality was assessed using eosin Y (CI 45380 Sigma- Aldrich, St. Louis, MO, USA) staining, evaluating a minimum of 300 spermatozoa per sample and distinguishing viable (unstained) from non-viable (red-stained) cells. The test is based on membrane integrity: live spermatozoa have intact plasma membranes that exclude the dye, whereas dead spermatozoa with damaged membranes allow the dye to penetrate and appear pink under the microscope. After semen evaluation, samples were centrifuged at 400× *g* for 15 min to separate seminal plasma from spermatozoa. After centrifugation, the seminal plasma was examined under a light microscope to ensure the absence of spermatozoa. The sperm pellet was washed three times in phosphate-buffered saline, and aliquots with around 2 million spermatozoa were prepared for other analyses. Both seminal plasma and spermatozoa were stored at −80 °C until use.

### 4.3. F_2_-Isoprostane Determination by ELISA Assay

Free F_2_-IsoPs were assessed by measuring 8-iso-prostaglandin F_2α_ (8-iso-PGF_2α_, hereafter referred to as 8-isoprostane) in seminal plasma using a commercially available ELISA kit (8-isoprostane ELISA Kit, Cayman Chemical, Ann Arbor, MI, USA). The assay is based on a competitive enzyme immunoassay employing a specific anti-8-isoprostane antibody, an 8-isoprostane–acetylcholinesterase (AChE) tracer, and Ellman’s reagent as the chromogenic substrate. Microplates pre-coated with a mouse anti-rabbit IgG capture antibody were used according to the manufacturer’s instructions. Absorbance was measured spectrophotometrically at 405 nm, and 8-isoprostane concentrations were calculated by interpolation from a standard curve generated using known concentrations of 8-isoprostane (range: 0.8–500 pg/mL). Each sample was analyzed in duplicate, and the results were expressed as pg/mL.

### 4.4. Nrf2 Determination by ELISA Assay

Nrf2 levels were quantified in seminal plasma and sperm cells. Briefly, spermatozoa were lysed in RIPA buffer supplemented with a protease inhibitor cocktail, and clarified lysates were collected after centrifugation and stored at −80 °C until analysis. Nrf2 concentrations were then measured using a commercially available ELISA (Human Nrf2 ELISA Kit, Cat. N. EH348RB, Invitrogen, Thermo Fisher Scientific, Waltham, MA, USA). For the assay, sensitivity was 14 pg/mL, and intra-assay and inter-assay coefficients of variation (CV) were <10% and <12%, respectively. The assay is based on a sandwich ELISA format employing microplates pre-coated with a monoclonal antibody specific for human Nrf2. The same volume (100 µL) of seminal plasma, sperm lysate, and standard was added to the wells to allow antigen binding, followed by incubation with a biotin-conjugated detection antibody and streptavidin–horseradish peroxidase. After washing steps to remove unbound components, tetramethylbenzidine substrate was added as the chromogenic reagent, and the enzymatic reaction was stopped with the stop solution. Absorbance was measured spectrophotometrically at 450 nm, and Nrf2 concentrations were calculated by interpolation from a standard curve generated using known concentrations of recombinant human Nrf2 (range: 14–10,000 pg/mL). Each sample was analyzed in duplicate using 50 µL per well, and results were expressed as pg/mL.

### 4.5. RNA Extraction and Quantitative Reverse Transcription PCR Analysis

RNA extraction and qRT-PCR were performed in a total of 24 randomly selected subjects, 6 for each group. Total RNA was isolated from ejaculated human spermatozoa using the PureLink^®^ RNA Mini Kit (Thermo Fisher Scientific, Waltham, MA, USA), following the manufacturer’s protocol. RNA yield and purity were determined spectrophotometrically by measuring absorbance at 260 and 280 nm with a NanoDrop™ One/OneC Microvolume UV–Vis Spectrophotometer (Thermo Fisher Scientific, Waltham, MA, USA). For complementary DNA synthesis, 500 ng of total RNA from each sample were reverse-transcribed using the High-Capacity cDNA Reverse Transcription Kit (Applied Biosystems, Waltham, MA, USA). qRT-PCR was subsequently performed to evaluate Nrf2, Keap1, Bach1, and HO-1 transcript levels in spermatozoa using specific PrimePCR™ SYBR™ Green assays (*NFE2L2* qHsaCED0038543; *KEAP1* qHsaCID0017511; *BACH1* qHsaCED0045718; *HMOX1* qHsaCID0022141; BioRad Laboratories, Inc., Hercules, CA, USA). Amplification reactions were carried out on a QuantStudio™ 5 Real-Time PCR System (Thermo Fisher Scientific, Waltham, MA, USA). Gene expression data were normalized against the housekeeping gene glyceraldehyde-3-phosphate dehydrogenase (*GAPDH*), assessed in parallel (PrimePCR™ SYBR™ Green Assay *GAPDH* qHsaCED0038674, Bio-Rad Laboratories, Inc., Hercules, CA, USA). No-template controls and reverse transcription–negative controls were included in each run to exclude contamination and genomic DNA amplification. The PCR cycling conditions consisted of an initial incubation at 50 °C for 2 min, followed by enzyme activation at 95 °C for 2 min, and 40 amplification cycles comprising denaturation at 95 °C for 15 s and annealing/extension at 60 °C for 1 min. Relative mRNA expression levels were calculated using the comparative Ct method (2^−ΔΔCt^), and all reactions were performed in triplicate.

### 4.6. Statistical Analysis

The distribution of the variables was preliminarily evaluated using the Kolmogorov–Smirnov test to assess data normality. Given the non-Gaussian distribution of the data, non-parametric statistical methods were applied. Relationships between variables were examined using Spearman’s rank correlation coefficient. Comparisons between two independent groups were carried out using the Mann–Whitney U test. When more than two groups were analyzed, differences among groups were initially assessed using the Kruskal–Wallis test, followed by Dunn’s post hoc test for pairwise comparisons. Results are reported as median values with corresponding interquartile ranges (IQR: 25th–75th percentiles). A two-tailed *p*-value < 0.05 was considered statistically significant. Statistical analyses were performed using GraphPad Prism (version 8.4.2).

## 5. Conclusions

Our findings indicate that the Nrf2 levels in human semen reflect a complex, compartment-specific response to OS rather than a cytoprotective mechanism. In particular, Nrf2 levels in spermatozoa could represent an adaptive activation during spermatogenesis in response to ROS that induce lipid peroxidation, whereas seminal plasma Nrf2 could more likely reflect oxidative damage occurring within the male reproductive tract, rather than a direct antioxidant defense.

## Figures and Tables

**Table 1:** Medians (IQR) of semen parameters, seminal F_2_-Isoprostanes (F_2_-IsoPs), and both seminal and sperm cell Nrf2 levels of the total group (79 subjects).

Variables	Median (25th–75th Percentiles)
Semen parameters	
Volume (mL)	3.60 (2.80–4.70)
Sperm ×10^6^/mL	42.50 (23.00–68.00)
Progressive motility (%)	34.00 (25.00–45.00)
Normal morphology (%)	8.00 (5.00–13.00)
Vitality (%)	67.00 (55.00–80.00)
Seminal plasma	
F_2_-IsoPs (pg/mL)	51.90 (29.76–62.66)
Nrf2 (pg/mL)	24.16 (14.40–40.84)
Spermatozoa	
Nrf2 (pg/mL)	101.73 (44.87–175.25)

**Table 2:** Correlations (rho, Spearman’s coefficient) between all considered variables in 79 individuals. Statistics are also reported: * *p* < 0.05, ** *p* < 0.01, *** *p* < 0.001.

	Volume (mL)	Sperm ×10^6^/mL	Progressive Motility (%)	Normal Morphology (%)	Vitality (%)	F_2_-IsoPs (pg/mL)	Seminal Plasma Nrf2 (pg/mL)	Spermatozoa Nrf2 (pg/mL)
Volume (mL)	1.000	0.112	0.159	−0.054	0.038	−0.150	−0.127	−0.183
Sperm ×10^6^/mL	0.112	1.000	0.404 ***	0.340 **	0.394 ***	−0.179	0.035	−0.171
Progressive motility (%)	0.159	0.404 ***	1.000	0.656 ***	0.716 ***	−0.419 ***	−0.163	−0.280 *
Normal morphology (%)	−0.054	0.340 **	0.656 ***	1.000	0.643 ***	−0.409 ***	0.019	−0.188
Vitality (%)	0.038	0.394 ***	0.716 ***	0.643 ***	1.000	−0.635 ***	−0.258 *	−0.371 ***
F_2_-IsoPs (pg/mL)	−0.150	−0.179	−0.419 ***	−0.409 ***	−0.635 ***	1.000	0.430 ***	0.518 ***
Seminal plasma Nrf2 (pg/mL)	−0.127	0.035	−0.163	0.019	−0.258 *	0.430 ***	1.000	−0.019
Spermatozoa Nrf2 (pg/mL)	−0.183	−0.171	−0.280 *	−0.188	−0.371 ***	0.518 ***	−0.019	1.000

**Table 3:** Medians and 25th–75th percentiles of semen parameters, levels of Nrf2 and F_2_-Isoprostanes (F_2_-IsoPs) in seminal plasma, and levels of Nrf2 in spermatozoa across different groups: fertile (F), varicocele (V), urogenital infections (UI), unknown fertility status, without known reproductive or systemic pathologies (UFS). Statistics are also reported.

	Fertile (F, *n* = 19)	Varicocele (V, *n* = 22)	Urogenital Infections (UI, *n* = 23)	Unknown Fertility Status (UFS, *n* = 15)	Statistics
Volume (mL)	4.20 (2.80–5.00)	3.50 (2.75–5.00)	3.40 (2.10–4.40)	4.00 (3.50–4.50)	ns
Sperm ×10^6^/mL	58.00 (42.00–80.00)	44.75 (21.00–79.25)	32.00 (13.60–54.00)	27.50 (8.00–55.00)	F vs. UI *p* < 0.01 F vs. UFS *p* < 0.05
Progressive motility (%)	55.00 (47.00–66.00)	35.50 (26.00–39.75)	28.00 (19.00–41.00)	24.00 (18.00–29.00)	F vs. V *p* < 0.001 F vs. UI *p* < 0.001 F vs. UFS *p* < 0.001
Normal morphology (%)	14.00 (13.00–15.00)	6.00 (5.00–10.50)	6.00 (5.00–8.00)	7.00 (4.00–9.00)	F vs. V *p* < 0.001 F vs. UI *p* < 0.001 F vs. UFS *p* < 0.001
Vitality (%)	88.00 (84.00–90.00)	65.00 (63.75–70.00)	55.00 (48.00–60.00)	68.00 (55.00–76.00)	F vs. V *p* < 0.001 F vs. UI *p* < 0.001 F vs. UFS *p* < 0.001
Seminal plasma F_2_-IsoPs (pg/mL)	26.43 (22.34–28.33)	61.33 (54.09–72.87)	57.14 (44.05–72.62)	45.24 (36.43–58.97)	F vs. V *p* < 0.001 F vs. UI *p* < 0.001 F vs. UFS *p* < 0.05 V vs. UFS *p* < 0.05
Seminal plasma Nrf2 (pg/mL)	23.58 (14.12–27.04)	38.04 (14.13–95.78)	31.25 (17.66–45.49)	16.30 (12.23–40.27)	ns
Spermatozoa Nrf2 (pg/mL)	38.80 (27.04–57.52)	142.70 (94.90–194.90)	183.40 (95.51–259.50)	88.31 (64.04–134.80)	F vs. V *p* < 0.001 F vs. UI *p* < 0.001 F vs. UFS *p* < 0.05

## Data Availability

The original contributions presented in this study are included in the article. Further inquiries can be directed to the corresponding author.

## Abbreviations

The following abbreviations are used in this manuscript:

OS	Oxidative Stress
ROS	Reactive Oxygen Species
F_2_-IsoPs	F_2_-Isoprostanes
WHO	World Health Organization
qRT-PCR	Quantitative Reverse Transcription Polymerase Chain Reaction
Nrf2	Nuclear Factor-E2-Related Factor 2
Keap1	Kelch-Like ECH-Associated Protein 1
sMAF	Small Musculoaponeurotic Fibrosarcoma
ARE	Antioxidant Response Element
Bach1	BTB and CNC Homology 1
HO-1	Heme Oxygenase-1
GAPDH	Glyceraldehyde-3-Phosphate Dehydrogenase
AChE	8-Isoprostane–Acetylcholinesterase
